# Novel near-diploid ovarian cancer cell line derived from a highly aneuploid metastatic ovarian tumor

**DOI:** 10.1371/journal.pone.0182610

**Published:** 2017-08-07

**Authors:** Ester Rozenblum, Jose R. Sotelo-Silveira, Gina Y. Kim, Jack Y. Zhu, Christopher C. Lau, Nicole McNeil, Susana Korolevich, Hongling Liao, James M. Cherry, David J. Munroe, Thomas Ried, Paul S. Meltzer, Walter M. Kuehl, Anna V. Roschke

**Affiliations:** 1 Genetics Branch, CCR, NCI, NIH, Bethesda, Maryland, United States of America; 2 Laboratory of Molecular Technology, Advanced Technology Program, SAIC-Frederick, Inc., NCI-Frederick, Frederick, Maryland, United States of America; 3 Advanced Technology Program, SAIC-Frederick, Inc., NCI-Frederick, Frederick, Maryland, United States of America; Philipps University, GERMANY

## Abstract

A new ovarian near-diploid cell line, OVDM1, was derived from a highly aneuploid serous ovarian metastatic adenocarcinoma. A metastatic tumor was obtained from a 47-year-old Ashkenazi Jewish patient three years after the first surgery removed the primary tumor, both ovaries, and the remaining reproductive organs. OVDM1 was characterized by cell morphology, genotyping, tumorigenic assay, mycoplasma testing, spectral karyotyping (SKY), and molecular profiling of the whole genome by aCGH and gene expression microarray. Targeted sequencing of a panel of cancer-related genes was also performed. Hierarchical clustering of gene expression data clearly confirmed the ovarian origin of the cell line. OVDM1 has a near-diploid karyotype with a low-level aneuploidy, but samples of the original metastatic tumor were grossly aneuploid. A number of single nucleotide variations (SNVs)/mutations were detected in OVDM1 and the metastatic tumor samples. Some of them were cancer-related according to COSMIC and HGMD databases (no founder mutations in *BRCA1* and *BRCA2* have been found). A large number of focal copy number alterations (FCNAs) were detected, including homozygous deletions (HDs) targeting *WWOX* and *GATA4*. Progression of OVDM1 from early to late passages was accompanied by preservation of the near-diploid status, acquisition of only few additional large chromosomal rearrangements and more than 100 new small FCNAs. Most of newly acquired FCNAs seem to be related to localized but massive DNA fragmentation (chromothripsis-like rearrangements). Newly developed near-diploid OVDM1 cell line offers an opportunity to evaluate tumorigenesis pathways/events in a minor clone of metastatic ovarian adenocarcinoma as well as mechanisms of chromothripsis.

## Introduction

Ovarian cancer continues to be a silent killer, asymptomatic until it is very advanced. Most women are diagnosed at stage III to IV, when the tumor has metastasized to peritoneal and distant organs. At this stage the treatment is extremely difficult since the tumors became resistant to the majority of cancer therapies. Among the fundamental priorities identified at the 2015 Ovarian Cancer Action meeting are development of genomically characterized ovarian cancer cell lines, and characterization of clonal heterogeneity and genome instability in acquired drug resistance disease [1]. With the recent progress in new ovarian cancer cell lines development [2], many more well characterized cell lines derived from ovarian cancers are still needed due to the high level of genomic heterogeneity among and within ovarian tumors [3, 4].

The aim of this study was to characterize OVDM1, a novel ovarian cell line derived from a stage IV serous ovarian adenocarcinoma from an Ashkenazi Jewish patient with early onset of disease. Characterization included cell morphology, genotyping, tumorigenic assay, mycoplasma testing, molecular profiling of the whole genome by aCGH and gene expression microarray, targeted sequencing of a panel of 197 cancer-related genes as well as SKY. Early passages of OVDM1 were compared with the corresponding metastatic tumor samples. Molecular characterization of progression events from early to late cell line passages was also performed.

## Materials and methods

### Tumor samples

Stage IV serous ovarian adenocarcinoma metastatic tumor samples from an ovarian cancer Ashkenazi Jewish patient with early onset (age 44) were obtained after a second cytoreductive surgery, three years after the first surgery removing the primary tumor, both ovaries, and the remaining reproductive organs, was performed. Primary cytoreductive surgery was followed by several systemic therapies utilizing Taxol, Carboplatin, Doxil and Gemzar. Three samples from the visceral peritoneal area were received after the second surgery: one was kept in serum and devoted to cell culture, and the two other metastatic tumor samples (MT) had been freshly frozen. Patient written consent was obtained as well as approval from the Office of Human Subject Research, NIH.

### Immortalization and cell culture

The metastatic tumor sample was mechanically disrupted and incubated in a special conditioned medium [5] supplemented with EGF 200 ng/ml and Hydrocortisone 0.4 μg/ml final concentration. After almost 3 months of primary culture, only two passages were obtained and the cells began to senesce. They were then transfected with SV40 large-T antigen and/or hTERT. Only the cells co-transfected with SV40 large-T antigen and hTERT continued growing and produced an immortalized cell line, called OVDM1.

OVDM1 was cultured in a medium, created by mixing equal volumes of MCDB 105 Medium and Medium 199 (both Sigma-Aldrich, St. Louis, MO)[5]. Heat inactivated FBS, 50 μg/ml of Gentamicin and 100X Antibiotic/Antimycotic (all Invitrogen/Life Technologies) were added to a final concentration of 5%, 50 μg/ml and 1X respectively. When close to confluence, cells were subcultered in a split ratio of 1 to 4. Different passages of OVDM1 cells were frozen in 7% DMSO, 40% heat inactivated FBS and 53% of the above medium.

### DNA and RNA

Genomic DNA and total RNA from MTs and three different passages of the immortalized ovarian cell line (P3, P18 and P30) were extracted with Qiagen Blood & Cell Culture DNA Midi Kit and Qiagen miRNeasy Mini Kit (Qiagen, Valencia, CA).

DNA from MTs and OVDM1 P3 and P30 was sent to LabCorp (https://www.labcorp.com/) for short tandem repeat (STR) genotyping. The 16 genetic markers used were: D8S1179; D21S11; D7S820, CSF, D3S1358. THO1, D13S317, D16S539, D2S1338, D19S433, rWA, TPOX, D18S51, D5S818, FGA, Amelogenin.

Mycoplasma contamination screening was performed utilizing the Venor GeM Mycoplasma Detection Kit, PCR based (Sigma-Aldrich, St. Louis, MO).

### SKY

We performed SKY on OVDM1 P3, P18 and P30 using commercial probes (Applied Spectral Imaging, Carlsbad, CA) according to manufacturer protocol. Image acquisition was conducted using a CCD camera connected to SD200 Spectracube (Applied Spectral Imaging) mounted on a Leica DMRXA microscope through a custom design optical filter (Chroma Technology, Bellows Falls, Vermont). Image acquisition was done with Spectral Imaging software and analysis with SKYView software (Applied Spectral Imaging).

### aCGH

Agilent CGH Microarray Kit 244A (G4411B) was used on three passages of OVDM1 and MT1(for MT2 aCGH 44B, G4410B was used), according to the Agilent protocol, with the exception that genomic DNA of the cell line or the tumor tissue was labeled with Cy3 (Jackson ImmunoResearch Laboratories, West Grove, PA) and reference normal human male genomic DNA (Promega, Madison, WI) was labeled with Cy5 (Jackson ImmunoResearch Laboratories). Data was extracted from array images with Agilent Feature Extraction Software.

Copy number alterations (CNAs) were determined with Agilent Genomic Workbench version 7.0.4.0 using Circular Binary Segmentation (CBS) algorithm [6, 7]; and with Nexus CN 6.1 utilizing the Fast Adaptive States Segmentation Technique (FASST2) algorithm. Chromosomes X and Y were excluded from the analysis.

### Gene expression profiling

Gene expression microarray analysis was performed on both MTs and OVDM1 P3 and P30 using Affymetrix Human Genome U133 Plus 2.0 Array. For RNA amplification, fragmentation, labeling and hybridization, Affymetrix GeneChip 3’IVT Express User Manual P/N 702646 Rev 6, was followed. For washing and staining, Affymetrix GeneChip Fluidics Station 450/240 User’s Guide P/N 08–0092 Rev G was followed. Scanning was done using the Affymetrix GeneChip Scanner 3000. All experiments were conducted with 3 technical replicas per sample. Data analyses were performed using BRB-ArrayTools Version 4.3.0 –Beta_3 [8]. Genes showing minimal variation across the set of arrays were excluded from the analysis. Genes whose expression differed by at least 1.5 fold from the median in at least 20% of the arrays were retained. We used hierarchical clustering to cluster the samples [9]. Gene annotation enrichment analysis and clustering were performed, with DAVID (http://david.abcc.ncifcrf.gov/) [10]

Hierarchical clustering using BRB-ArrayTools was applied to a group of samples that included OVDM1 passages P3, and P30, and MTs, together with 767 samples comprised of 9 different tumor types including breast, cervix, colon (only adenomas), liver, lung, ovary, prostate, testicular and thyroid tumors and tumor cell lines; and a group of diseased non-cancerous endometrial tissue samples. Normal samples were also present among breast, colon, ovary and thyroid samples. CEL files corresponding to the HG_U133_Plus-2 platform were downloaded from the Gene Expression Omnibus (GEO) database (http://www.ncbi.nlm.nih.gov/geo/).

### Mutational analysis

Extracted DNA (500 ng) was fragmented by sonication using a Covaris S2 Focused ultrasonicator to a mean size of approximately 300 bp. These DNA fragments were end-repaired and phosphorylated with T4 DNA polymerase, Klenow fragment, and T4 polynucleotide kinase (all from New England Biolabs, Ipswich, MA). An A-base overhang was introduced to the 3’ ends of the fragments with Klenow exo-minus (New England Biolabs) and these fragments were ligated to Illumina paired-end adaptors with T4 DNA Ligase (Enzymatics, Beverly, MA). The adapter ligated fragments with a mean size of 350 bp +/- 20% were purified using the Agencourt AmpureXP Purification System (Beckman Coulter, Indianapolis, IN) and amplification was performed using Illumina PCR primers InPE1.0 and InPE2.0 and primer indices. Pooled, indexed libraries were captured using an Agilent SureSelect Custom DNA kit targeting exons of 197 commonly mutated cancer genes (Agilent Technologies, Santa Clara, CA) according to the manufacturer’s protocol. Sequencing was done on Illumina’s Miseq sequencers and further sequencing data processing and analysis was done with the in-house developed processing and variant calling pipelines. Briefly, after comprehensive QC analysis, the raw sequencing reads were mapped to human genome build 19 by Burrows-Wheeler Aligner (BWA, Li and Durbin, 2009) and variant calling was subsequently performed by the mpileup program in the Samtools software (http://samtools.sourceforge.net/). Then the variants, including SNVs and Indels, were annotated with the Annovar software (http://www.openbioinformatics.org/annovar/). Finally, manual inspection was done on the clinically relevant genes in the Integrative Genomics Viewer (IGV, http://www.broadinstitute.org/igv/).

### Xenograft assay

Early and late passages of OVDM1 were injected into athymic NCr Nu/Nu mouse strain (Frederick National Laboratory for Cancer Research, Frederick, MD). The athymic NCr Nu/Nu mice were 6–8 weeks old. Mice were anesthetized with 0.20% Ketamine administered by intraperitoneal injection (Vedco Inc., Saint Joseph, MO). Cells (3x 10^6^) were injected subcutaneously above the left shoulder of each mouse (4 females, 2 male). Cells (3x10^6^) suspended in a mixture of 200ul of 1x PBS and/or 100ul of Matrigel Basement Membrane (BD, Franklin Lakes, NJ) were injected over the right shoulder of each mouse. The nude mice were monitored over the course of 52 weeks to determine tumor development. All animals were handled and euthanized following animal protocols (NIH Study Animal Study Protocol MB-045).

## Results

**Fig 1:**
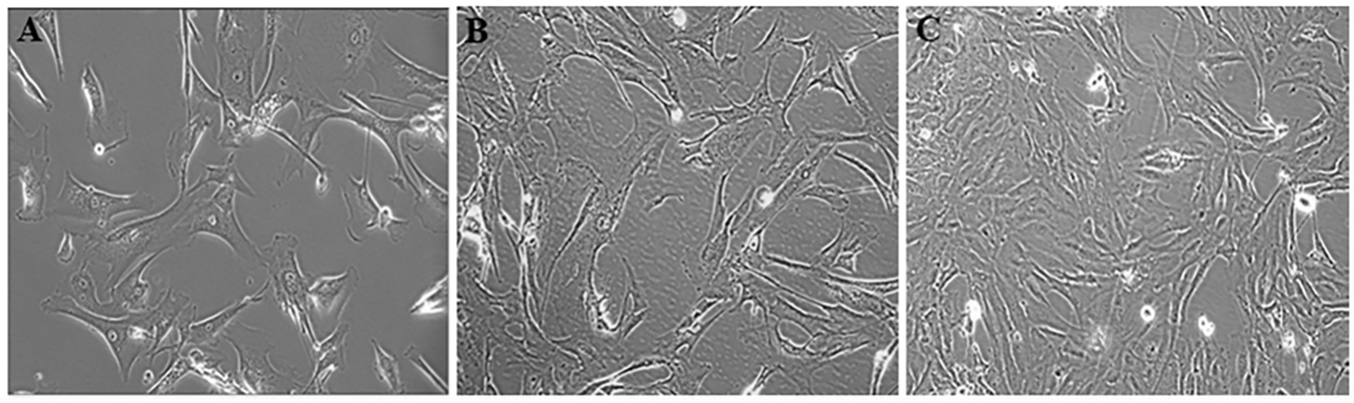
OVDM1 ovarian cancer cell line derived from an ovarian metastatic tumor after immortalization with SV40 large-T antigen and hTERT. (A) Primary culture of the ovarian metastatic tumor prior to immortalization. (B) Early passage of OVDM1 after immortalization. (C) Late passage of OVDM1 after immortalization.

Cells in primary culture and early passages were large and polygonal shaped. Cells in later passages were smaller and more fibroblast-like (Fig 1). OVDM1 cells at early and late passages were Mycoplasma negative and shared the same genotype with original tumor sample MT1.

To confirm the ovarian origin of OVDM1, gene expression microarray data of early and late passages were clustered by hierarchical clustering with a broad group of samples corresponding to different tumor types and normal tissues (see Materials and Methods). The OVDM1 (P3 and P30) clearly clustered together with a group of ovarian normal tissue, cancers, and cell lines, but not with other tumors and tissues (S1 Fig).

The metastatic tissue and OVDM1 early and late passages were screened for mutations in the 197 cancer-related genes. Neither founder mutations in *BRCA1* (185delAG, 5382InsC) and *BRCA2* (6174delT) were present. On the other hand, 5 SNVs were found in *BRCA1* gene and one SNV associated with breast cancer risk in *BRCA2* (S1 Table).

No tumors developed in the nude mice after the injection of early or late passages of the OVDM1 cell line during 52 weeks, in the xenograft assay.

### Characterization of the early passage of OVDM1

**Fig 2:**
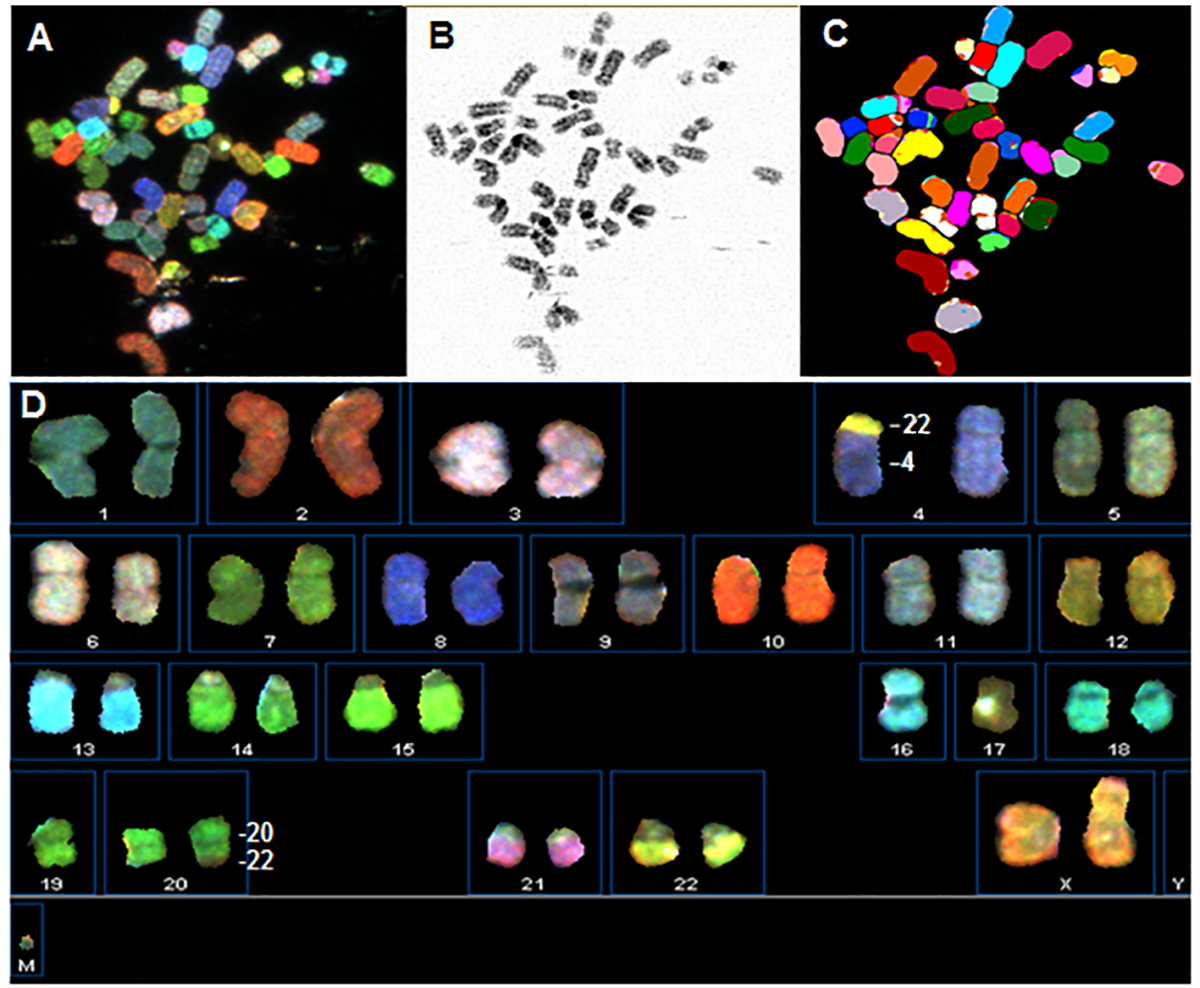
OVDM1 early passage karyotype: 42–47, XX, der(4)t(4;22)(p11;q11),-16, der(22)t(20;22)(q11?,q13). Upper panel shows a metaphase spread of OVDM1-P3: (A) chromosomes in display colors, (B) G-banding-like DAPI converted image, (C) chromosomes in classification colors. Lower panel shows SKY karyotype: (D) classified chromosomes from the same metaphase in display colors.

**Fig 3:**
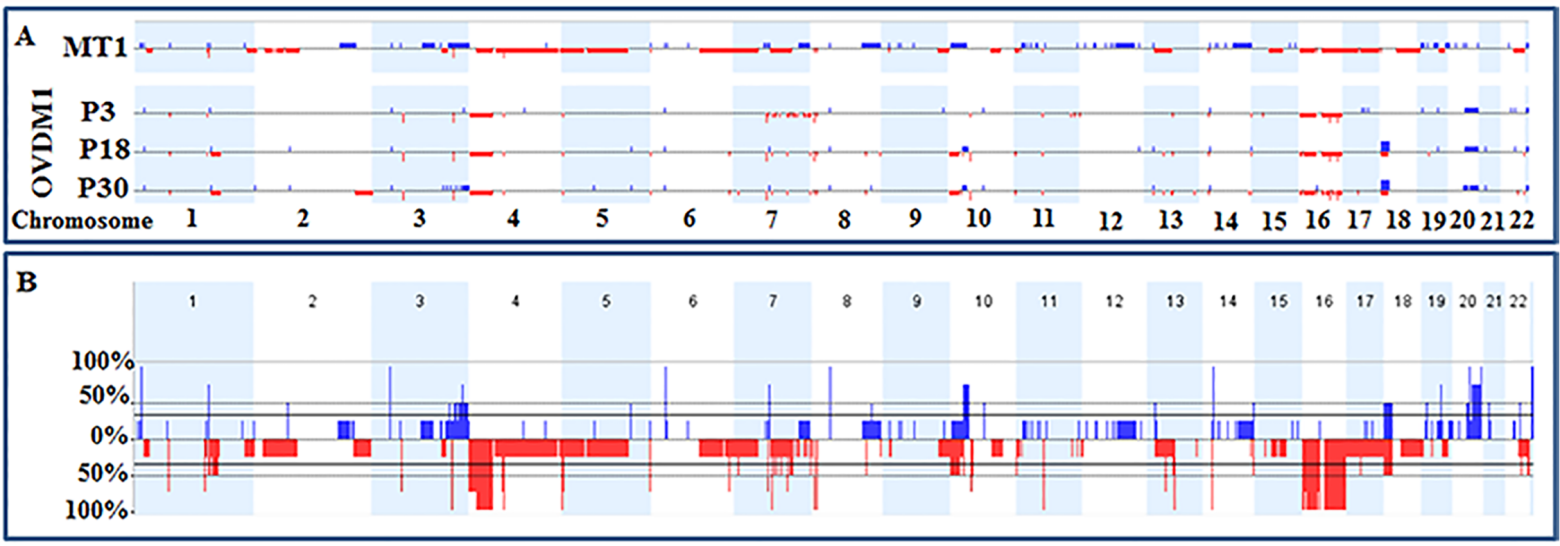
Whole genome view of CNAs in an ovarian metastatic tumor sample and the newly established OVDM1 ovarian cancer cell line, derived from the same tumor. (A) Whole genome CNA profiles of MT1 sample and OVDM1 cell line. (B) Frequency of CNAs considering MT1 and early, middle and late passages of OVDM1. Blue lines–gains, red–losses.

OVDM1-P3 has a near-diploid karyotype with low-level aneuploidy (Fig 2): loss of one chromosome 16 and two unbalanced translocations, der(4)t(4;22)(p11;q11) (all cells) and der(22)t(20;22)(q11?,q13) (50% of cells), resulted in the loss of 4p and gain of a part of 20q. aCGH confirmed the above whole chromosome or chromosome arm alterations and revealed additional small (<10Mb) focal losses and gains (FCNA) (Fig 3). Altogether 71 FCNAs were identified in OVDM1- P3, 45 losses and 26 gains, most of them were several hundred Kb long (Table 1, S2 and S3 Tables). Among them, 18 losses and 10 gains overlapped with common CNV areas. Usually, 0–5 small focal losses and gains per chromosome were present, with the exception of chromosome 7 where 21 small focal losses were detected (18 of them in q22.1-q36.3 area) and 20q with 11 focal gains.

**Table 1:** Whole chromosome gains and losses and number of FCNAs in tumor sample MT1 and OVDM1 cell line (early, intermediate, and late passages).

Sample	Whole chromosome gains and losses	# of focal CNAs						
		Total #	Gains	Large gains >10Mb	Small gains <10Mb	Losses	Large losses >10Mb	Small losses <10Mb
**MT1**	-16, -17	154	77	10	67	77	26	51
**OVDM1-P3**	-16	71	26	1	25	45	1	44
**OVDM1-P18**	-16	161	73	2	71	88	2	86
**OVDM1-P30**	-16	186	88	2	86	98	3	95

Six HDs were found among FCNAs present in OVDM1-P3. The HDs’ locations and their targets were: 3p14.2 (*FHIT*, CNV area), 3q26.1 (transcript BC073807, CNV area); 7q11.2 (*AUTS2*); 8p23.1 (*FAM90A*, defensins, *GATA4*, CNV area); 16q21 (no genes); and 16q23.1 (*WWOX*, CNV area) (S2 Table). The HDs’ targeting genes were all intragenic.

Sequencing of 197 cancer-related genes identified the presence in OVDM1-P3 of five SVs: one frameshift deletion (*NOTCH2*), a variant in the splicing site (*MSH2*), and three exonic non-synonymous SNVs/mutations (in *PBRM1*, *RUNX1*, *HNF1A*) in addition to 63 exonic non-synonymous SNVs common for OVDM1-P3 and MT1 (Table 2 and S1 Table). All additional SVs were heterozygous.

**Table 2:** Summary of sequence variants (SV) found in 197 cancer-related genes in tumor sample MT1 and OVDM1 cell line.

	SV found in 197 cancer-related genes				
	Total # of variants	Exonic			Splicing area
		Frameshift	Non-synonymous	Synonymous	
**MT1**	222	1	69	152	0
**OVDM1-P3**	221	1	66	153	1
**OVDM1-P30**	222	0	69	152	1
**# of SVs common for MT1, P3 and P30**	212	0	63	149	0
**Total # of common and uncommon SVs**	234	2	74	156	2

### Metastatic tumor samples and comparison with OVDM1-P3

Two frozen samples from the metastatic site (MT1 and MT2) showed the same highly aneuploid aCGH profiles. MT1 aCGH profile is shown on Fig 3A. Similar to P3, loss of chromosome 16 and 4p was observed in MTs, but with retention of 4p15.32-pter. In addition, there was a loss of the whole chromosome 17 and chromosome arms 4q and 5p, and 36 other large FCNAs (>10Mb) (Table 1). Only MT1 was used for FCNA analysis due to higher probe coverage (see Materials and Methods). The total number of the large and small FCNAs in MT1 was 154, comprised of 77 losses and 77 gains (Table 1). Among them, 10 focal losses and 23 gains were associated with common CNV areas (S2 Table). From 0 to 6 losses and from 0 to 9 gains per chromosome were observed, with exceptions of chromosome 2 where 25 focal losses were detected (24 of them clustered in 2p24.2-p11.2 area) and chromosome 17 where 15 losses were found (S2 Table).

Several FCNAs in MT1 overlapped with the FCNAs present in OVDM1-P3: 10 areas of genomic loss and 10 areas of gains (75% of them overlapped also with common CNV areas). Overlapped areas that have not been associated with common CNVs included one loss (16q11-q24.3) and four gains (3p22.2, 3q26.2-q29, 19p13.2, 20q13.33). Among three HDs found in MT1, only one was also homozygously deleted in the P3: HD in 3q26.1 targeting transcript BC073807 (S2 Table). Two other HDs were located at 1q21.3 (*LCE3C* gene) and 4q13.2 (*UGT2B17*, *UGT2B15*). In addition, allelic losses of 8p23.1 (*GATA4*), 16q21 (no genes) and 16q23.1 (*WWOX*) detected in MT1 may point to the existence of HDs in these areas similar to OVDM1.

Sequencing of 197 cancer-related genes in MT1 revealed one frameshift deletion (in *TP53*) and four exonic non-synonymous SNVs/mutations (*DPYD*, *NOTCH1*, *GRP*, *PDZD4*) present in MT1 only compared with OVDM1-P3 and P30 (Table 2 and S1 Table).

Gene expression analysis identified 2438 genes expression of which was similarly changed in MTs and OVDM1-P3 samples compared to normal ovarian cell lines and tissues (S4 Table). GO term analysis revealed statistically significant enrichment of genes involved in multiple cancer-related pathways and many other pathways and biological processes implicated in tumorigenesis (S5 Table).

### Late passages of OVDM1

SKY analysis of OVDM1-P18 and OVDM1-P30 showed the same unbalanced translocations as in early passage P3. In addition, der(X;10)(q10;q10), der(14)t(14;16)(q32;q12) and der(16;18)(p10;p10) were found. aCGH of P18 and P30 showed loss of chromosome 16 and 4p, and gain of 20q. Many small FCNAs present in P3 (except multiple losses on 7q21.1-q36.3) were also present in the late passages (S2 Table). Total number of FCNAs increased from 71 in P3 to 161 in P18, and to 186 in P30 (Table 1). Many changes occurred on 16p and 18p (82 gains and losses altogether), 1q23.1-q25.2 (14 losses in P18), 3q26.2-3q29 (14 gains in P30), 16q11.2-q24.3 (16 gains in P30) (S2 Table). OVDM1-P18 and P30 retained the same HDs as the early passage P3, and acquired two additional HDs, both inside the *FHIT* gene.

Four additional exonic non-synonymous SNVs/mutations in the sequenced cancer-related genes were detected in OVDM1-P30 compared to OVDM1-P3. Two of these SNVs had already been found in MT1 (S1 Table).

## Discussion

We have established and characterized a new ovarian near-diploid non-tumorigenic cell line OVDM1 derived from a stage IV serous ovarian adenocarcinoma metastatic tumor from an Ashkenazi Jewish patient after a second cytoreductive surgery. The second surgery was performed three years after the first surgery that removed the primary tumor, both ovaries, and the remaining reproductive organs.

Characterization of OVDM1 included SKY, aCGH, gene expression and sequencing of 197 cancer-related genes. The same procedures (excluding SKY) were applied to MTs. The newly developed cell line was considered ovarian because hierarchical (unsupervised) clustering of gene expression data clearly grouped the cell line with many other ovarian tumors, cell lines and ovarian normal samples, but not with tumors and tissues of different origin.

Early passage of OVDM cell line (P3) is near-diploid with loss of chromosome 16, one large FCNA and 71 small FCNAs, but two samples of the original metastatic tumor exhibit grossly aneuploid aCGH profiles with losses of chromosomes 16 and 17, 36 large FCNAs and 138 small FCNAs (Table 1). The presence of near-diploid precursors of OVDM1 in the site of highly aneuploid metastasis reflects genomic heterogeneity in the metastatic tumor. Genomic heterogeneity is evidenced by the coexistence of multiple different clones and is well documented in many tumor types [11]. Tumor polyclonality was first recognized by karyotypic studies in skin, head and neck, breast and pancreatic cancers among others [12–15]. Gorunova et al. [14] observed near-diploid clones in 40% of the pancreatic cancers studied; these clones showed simple numerical or structural chromosomal changes and colocalized with highly aneuploid tumor cells. More recently, next generation sequencing studies also found widespread presence of genomic heterogeneity in tumors, based on mutation and chromosomal aberration analyses [16]. Interestingly, the coexistence of near-diploid and highly aneuploid cells was found both in a primary and in a paired metastatic tumor [16]. Ovarian cancer disseminates mainly via extension of cancer cells from the primary tumor into intra-abdominal cavity. They travel as single cells or as multicellular aggregates to form secondary lesions [17]. OVDM1 could represent near-diploid ovarian cancer cells that traveled and further coexisted with the highly aneuploid cells at the site of the metastasis. Such coexistence also implies that the cells, from which OVDM1 originated, represent a side clone which emerged from the main clone before the latter cells became grossly aneuploid. Therefore, both clones may share mutations/rearrangements present at the early stages of carcinogenesis.

Indeed, a number of genetic variations/alterations were shared by OVDM1 cell line and MT1. First of all, they shared the vast majority (94%) of exonic synonymous and non-synonymous SNVs found in the set of 197 sequenced cancer-related genes. Due to the lack of normal tissue samples it was not possible to distinguish between germline and somatic SNVs. However, since many of these SNVs are present in high frequency in the normal human population, they most likely represent germline variants. Disregarding the origin, we tried to evaluate the significance of these SNVs for cancer development based on their presence in ovarian or other cancer samples through the search of COSMIC and HGMD databases. Ten SNVs were found to be associated with cancer risk in other studies: SNVs in *PGR* were associated with ovarian cancer risk, in *BRCA*2 was associated with breast cancer risk (even it was not a founder mutation), in *CYP1A1* and *ERBB2* were implicated in lung cancer risk, in *MSH6*, *MLH1* and *DCC* could increase risk of colorectal cancer, SNV in *PTCH1* was associated with increased risk of skin cancer, and synonymous change of *SMARCB1* was associated with hematopoietic, lymphoid and CNS malignancies. In addition, a small fraction of shared SNVs with a very low frequency in the normal population could include *de novo* mutations in cancer-related genes (S1 Table).

Gene expression analysis also revealed many similarities between OVDM1 and the corresponding metastatic tumor samples. Class comparison of OVDM1-P3 and MTs with a large group of normal ovarian tissue samples and cell lines revealed differential expression of 2438 genes. GO term analysis of this set of genes showed a statistically significant enrichment of genes involved in multiple cancer related pathways and biological processes implicated in tumorigenesis, such as regulation of cell cycle, apoptosis, cellular response to stress, regulation of cell proliferation, chromosome and cytoskeleton organization, DNA repair, nuclear transport, chromatin assembly and remodeling, DNA packaging, chromosome segregation, DSB repair, spindle organization, among others (S5 Table). Moreover, several of the shared cancer-related or low-frequency SNVs/mutations identified by sequencing, were involved upstream in the cancer-related pathways, identified by gene-expression analysis (S2 Fig), increasing the possibility that these SNVs/mutations were relevant to tumorigenesis in this case.

In contrast with multiple shared SNVs, only a few SNVs/mutations were present in the metastatic tissue or in the cell line only. Among five mutations exclusively present in MT1, *TP53* frameshift was associated with ovarian cancer, large intestine and upper autodigestive tract tumors (COSMIC database) and Li-Fraumeni syndrome (HGMD). These findings are illuminating given that samples of the metastatic tumor exhibit gross aneuploidy. This *TP53* frameshift found in the metastatic tissue could be responsible for allowing precursor cells to become highly aneuploid and progress to a major metastatic clone.

In contrast with SNVs, only 20 (10%) of FCNAs found in MT1 and OVDM1-P3 overlapped (S2 Table). Among the shared FCNAs was only one homozygous deletion (on 3q26.1). Two other HDs found in OVDM1-P3 involving *WWOX* and *GATA4* genes, had also been detected as allelic losses in MT1, and may point to the existence of HDs in these areas similar to OVDM1 (HDs are detected more readily in cell lines than in tissue due to contamination with normal cells).

**Fig 4:**
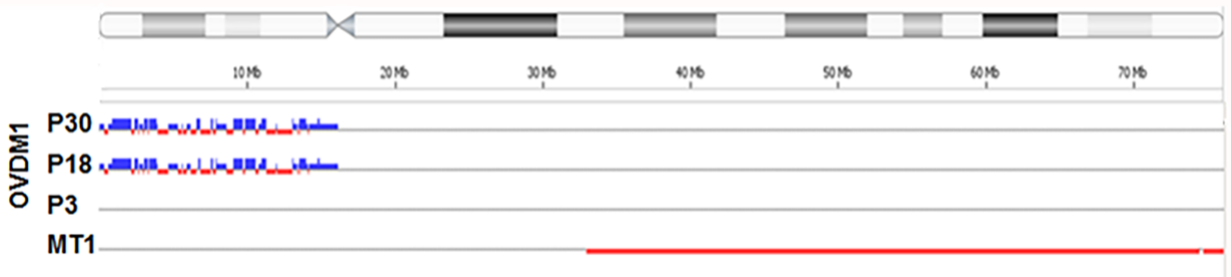
Chromosome 18 CNA profiles showing acquisition of chromothripsis-like rearrangements on 18p during cell culturing of OVDM1. Blue lines–gains, red–losses.

The vast majority of FCNAs (90%) were not shared between OVDM1-P3 and MT1 (S2 Table). Some of these focal CNAs were randomly distributed along chromosomes, others were clustered. During *in vitro* culturing, the OVDM1 cell line acquired additional genomic changes compared to the early passage: few gross chromosomal rearrangements and more than 100 new FCNAs. Remarkably, most of the newly acquired FCNAs were clustered and seems to be related to chromothripsis-like rearrangements (Fig 4).

Chromothripsis is a form of genomic instability characterized by multiple locally clustered breakpoints on a single chromosome or on a few chromosomes [18]. Our findings indicate that chromothripsis was operative in the newly developed cell line OVDM1. Since clustering of FCNA was observed in the MT1 also (S2 Table), this type of genomic instability perhaps operated in the major metastatic clone as well. This points to a possibility that major and minor clones with different combinations of genomic/chromosomal instability may coexist at the metastatic site. Newly developed near-diploid OVDM1 cell line offers an opportunity to evaluate tumorigenesis pathways/events in a minor clone of metastatic ovarian adenocarcinoma as well as mechanisms of chromothripsis.

## Supporting information

S1 Table Sequence variants (nonsynonymous, frameshift and splicing) found in MT1 and three different passages of OVDM1 by sequencing of 197 cancer-related genes. (XLSX)

S2 Table Chromosome areas of loss and gain. (XLSX)

S3 Table Size of focal copy number alterations. (PDF)

S4 Table Genes identified by BRB-ArrayTools class comparison of MT1 and OVDM1-P3 vs Ov Norm. (XLSX)

S5 Table Gene annotation enrichment analysis. Genes were identified by BRB-ArrayTools class comparison of MT1 and OVDM1-P3 vs normal ovarian samples and cell lines.
(XLSX)

S1 Fig Subset of the cluster of OVDM1 samples (early and late passages) and 767 other samples of normal, diseased (other than cancer), benign tumors and cancer samples from ten different tissue types, showing that OVDM1 groups with normal and cancer ovarian samples. (PPTX)

S2 Fig SNVs/mutations identified by sequencing in MT1 and OVDM1 and involved in the cancer-related pathways identified by gene-expression analysis. Genes with SNVs/mutations are labeled with black stars. Genes identified as differentially expressed in MT1 and OVDM1 compared to normal ovarian samples and cell lines are labelled with red stars.
(TIF)
