# Supplementary material for: Novel near-diploid ovarian cancer cell line derived from a highly aneuploid metastatic ovarian tumor
Source: PLoS One. 2017 Aug 7;12(8):e0182610. doi: 10.1371/journal.pone.0182610 (PMC5546722; doi:10.1371/journal.pone.0182610)
Supplement: S3 Table — (PDF) [file pone.0182610.s003.pdf]

S3 Table. Size of focal copy number alterations.

Sample	FCNA size					
	Focal gains (range)	Large gains >10Mb (average/median)	Small gains <10Mb (average/median)	Focal losses (range)	Large losses >10Mb (average/median)	Small losses <10Mb (average/median)
**MT1**	38Kb-40.1Mb	28,065Kb/30,387Kb	674Kb/170Kb	29Kb-86.7Mb	28,086Kb/21,181Kb	2,148Kb/1,051Kb
**OVDM1-P3**	46Kb-14.9Mb	14,860Kb	451Kb/177Kb	55Kb-48.9Mb	48,910Kb	468Kb/300Kb
**OVDM1-P18**	30Kb-17.5Mb	13,953Kb	335Kb/252Kb	26Kb-48.9Mb	31,062Kb	608Kb/287Kb
**OVDM1-P30**	30Kb-17.4Mb	14,723Kb	475Kb/248Kb	26Kb-48.9Mb	30,616Kb/22,553Kb	850Kb/289Kb
