# Supplementary material for: Novel near-diploid ovarian cancer cell line derived from a highly aneuploid metastatic ovarian tumor
Source: PLoS One. 2017 Aug 7;12(8):e0182610. doi: 10.1371/journal.pone.0182610 (PMC5546722; doi:10.1371/journal.pone.0182610)
Supplement: S1 Fig — (PPTX) [file pone.0182610.s006.pptx]

## Slide 1
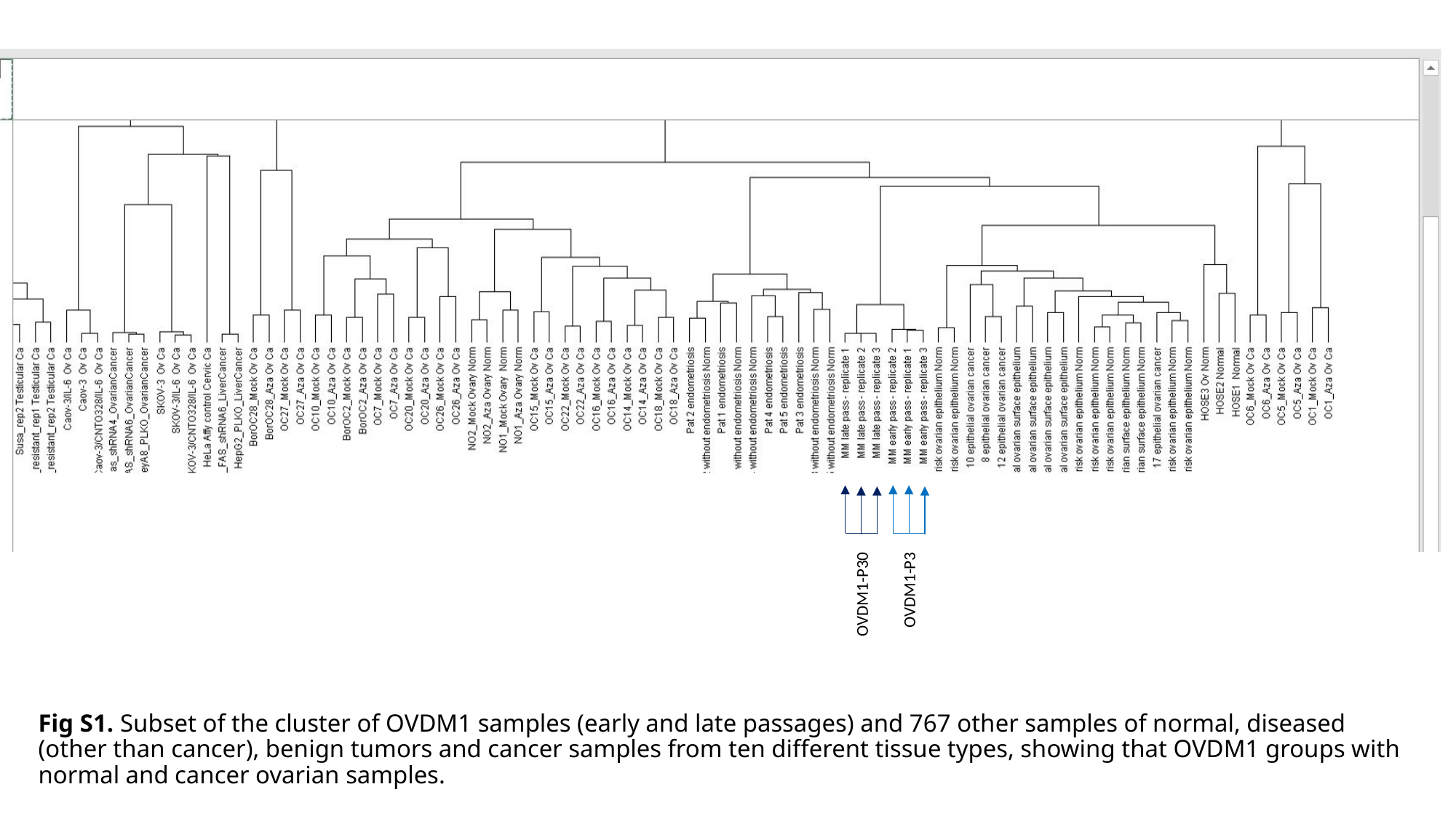

OVDM1-P3
OVDM1-P30
# Fig S1. Subset of the cluster of OVDM1 samples (early and late passages) and 767 other samples of normal, diseased (other than cancer), benign tumors and cancer samples from ten different tissue types, showing that OVDM1 groups with normal and cancer ovarian samples.
