# Supplementary figures and images for: Novel near-diploid ovarian cancer cell line derived from a highly aneuploid metastatic ovarian tumor
Source: PLoS One. 2017 Aug 7;12(8):e0182610. doi: 10.1371/journal.pone.0182610 (PMC5546722; doi:10.1371/journal.pone.0182610)

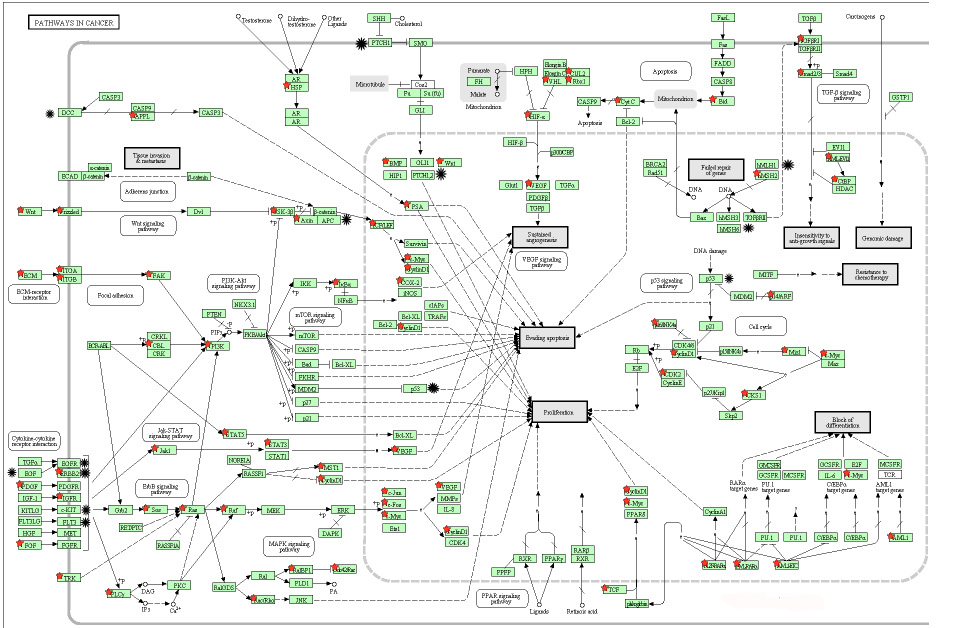

Supplement: S2 Fig — Genes with SNVs/mutations are labeled with black stars. Genes identified as differentially expressed in MT1 and OVDM1 compared to normal ovarian samples and cell lines are labelled with red stars. (TIF) [file pone.0182610.s007.tif]
